# A Relaxation App (HeartBot) for Stress and Emotional Well-Being Over a 21-Day Challenge: Randomized Survey Study

**DOI:** 10.2196/22041

**Published:** 2021-01-29

**Authors:** Laya Iyer, Ranjani B Iyer, Vetriliaa Kumar

**Affiliations:** 1 Code for Nonprofit Inc Novi, MI United States; 2 Heartfulness Program for Schools Heartfulness Institute Novi, MI United States; 3 Code for Nonprofit Inc Austin, TX United States

**Keywords:** Heartfulness, stress management, iOS app, social-emotional, mental health

## Abstract

**Background:**

HeartBot is an app designed to enable people 14 years and older to use relaxation tools offered by Heartfulness Institute to deal with daily stress and anxiety in a healthy, productive manner. These tools have proven effective in stress management and mental wellness when administered in a controlled environment by a certified proctor.

**Objective:**

This study aimed to explore the app’s effectiveness and evaluate the implementation of the tools.

**Methods:**

In this study, 88 participants were recruited and randomly sorted into 2 groups, the HeartBot intervention group (n=46) and the waitlist control group (n=42). Pre- and postsurveys measured participants’ stress levels using the Perceived Stress Scale (PSS) and their social-emotional well-being using the EPOCH (Engagement, Perseverance, Optimism, Connectedness, and Happiness) Measure of Adolescent Well-Being before and after they used the app for 21 days for 30 minutes every day.

**Results:**

The study received institutional review board approval on August 18, 2019. Participant recruitment lasted from the approval date until September 30, 2019. The 21-day challenge started on October 1, 2019. Of the 135 people who signed up, 88 completed the study. There was a statistically significant difference in the mean PSS scores before and after the intervention (from 18.3 to 7.89; *P*<.001). The paired Wilcoxon rank sum test on the EPOCH scores indicated a significant difference in the medians of the total scores (*W*=411.5, *P*<.001).

**Conclusions:**

Evidence from this study shows that HeartBot is an effective app that can be used to manage stress and improve positive characteristics of emotional wellness. Future research and widespread usage of the app under this study are encouraged based on this preliminary evidence of its effectiveness.

**Trial Registration:**

ClinicalTrials.gov NCT04589520; https://clinicaltrials.gov/ct2/show/NCT04589520

## Introduction

Stress is a major health problem around the world and is one of the main causes of early death and disease in the United States [1]. According to statistics from the American Institute of Stress [2], adults experience stress that is mainly caused by job pressure, money, health, relationships, poor nutrition, media overload, and sleep deprivation. About 77% of adults regularly experience physical symptoms caused by stress, 73% regularly experience psychological symptoms caused by stress, and 33% report living with extreme stress. Research shows that nearly half (49%) of all students report feeling a great deal of stress on a daily basis, and 31% report feeling somewhat stressed [3]. Childhood and adolescence are crucial formative developmental stages that lay the groundwork for an individual’s capacity to maintain their emotional well-being and mental health in adulthood [4,5]. These statistics show the urgent need for accessible stress management techniques that are effective for individuals of all ages.

Technology, especially in a portable form such as an app, is unique in introducing practical, accessible mental wellness tools to members of the general population. A previous study examining the effectiveness of mindfulness apps in improving users' well-being found that mindfulness-based positive intervention can be delivered via a smartphone app successfully [6]. Additionally, a study examining the effect of mindfulness apps versus traditional intervention stress techniques found that participants that used the app showed marginally more compassion satisfaction and marginally less burnout [7]. Relative to in-person interventions, digital technologies can reach a broader audience in less time, are cost-effective, and are more personalized to the individual [8].

There is a wide variety of apps that provide users with the tools to manage their stress, including apps such as Headspace and Calm. Such apps represent a convenient and cost-effective technology that can easily be scaled up to address barriers to implementing traditional mindfulness-based stress reduction programs but may require supplemental support to promote their use [9].

Digital mediums, therefore, have incredible potential for improving public health [10]. In addition, those using the app Calm for 8 weeks reported significant differences in outcomes of stress, mindfulness, and self-compassion [11]. This study aims to explore a stress management app to mitigate stress and promote overall well-being.

Heartfulness is a heart-based practice of meditation that focuses on the relationship between the heart and mind. It promotes further discovery in the science of yoga, as it relates to the body-mind complex and plays a vital role in expanding upon mindfulness practice. It is a journey to the center of the heart, a place of inner silence. Tuning into the heart develops calmness from within and uncovers every individual's brilliant self.

**Figure 1:**
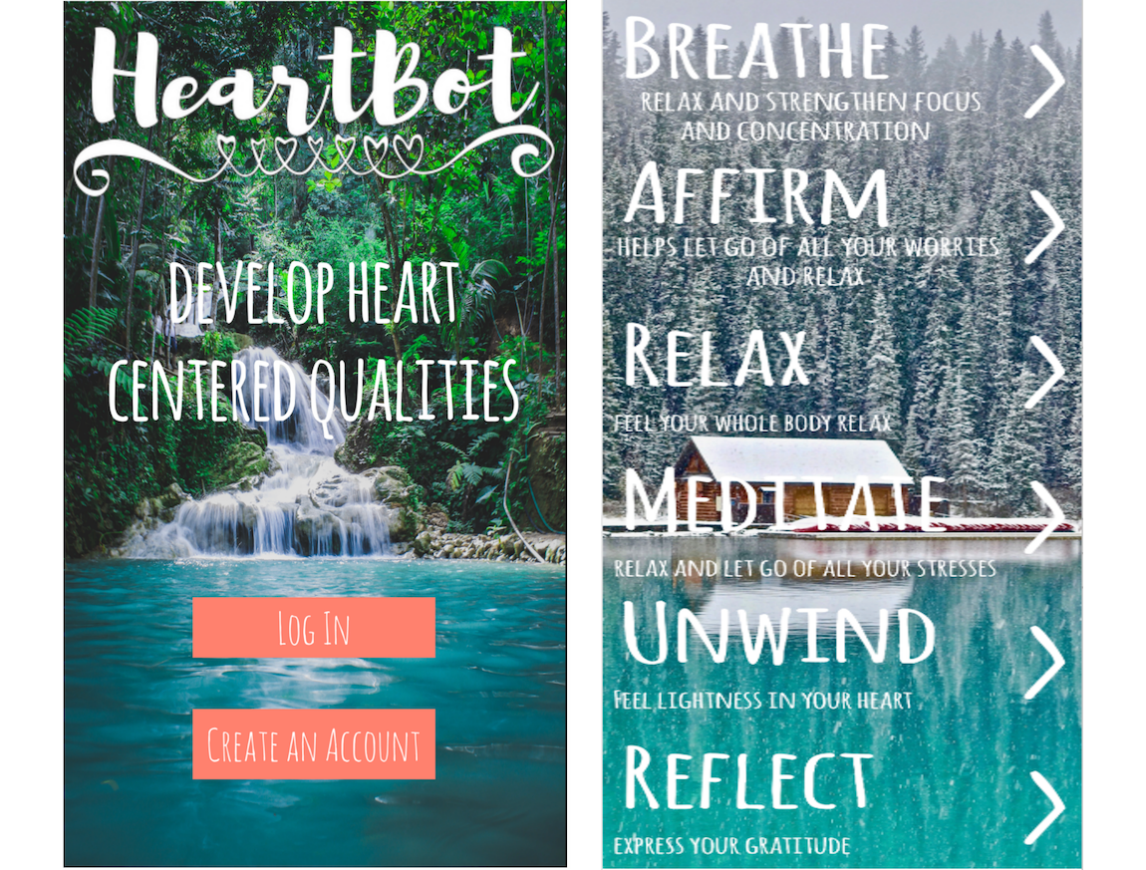
HeartBot app.

HeartBot, an iOS app, aims to provide its users with a convenient means of managing stress by providing personalized audios and guided experience of Heartfulness relaxation tools. Figure 1 presents the user interface of HeartBot, and the app features 6 guided tools. Previous studies concerning Heartfulness programs have established Heartfulness tools’ effectiveness in a structured environment [12]. This study expands upon previous research and investigates whether the HeartBot app is associated with a decrease in stress levels and an increase in emotional well-being in a 21-day challenge.

## Methods

### Study Design and Setting

This study used a pre- and postsurvey completed online. The intervention was completed remotely in a virtual setting.

### Ethics Approval

The study was approved by Solutions IRB (registration No. IORG0007116) in August 2019. All participants provided electronically signed consent and assent prior to participation in the study.

### Recruitment

**Figure 2:**
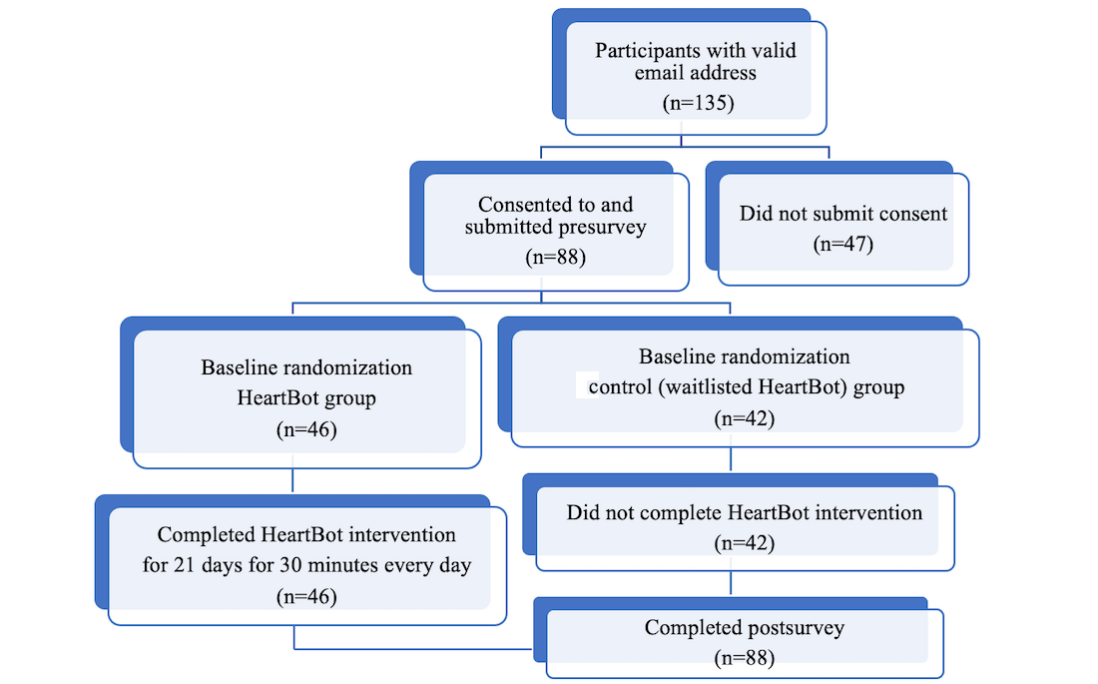
Study flowchart.

The participants’ recruitment was through convenience and snowball sampling because of their convenient accessibility and proximity to the researchers [13]. Posters were placed in schools and venues such as libraries to raise awareness about the study. Inclusion criteria to participate in the study were (1) being 14 years or older and (2) having an iOS device that allowed the user to download and use the app. Recruitment occurred from August 2019 through September 2019. We anticipated about 100 participants and expected a dropout rate of about 50%. We got responses from 135 interested participants, 88 of whom continued with the study and 47 of whom did not submit their consent. Figure 2 shows the study flowchart.

### HeartBot App and Intervention

**Figure 3:**
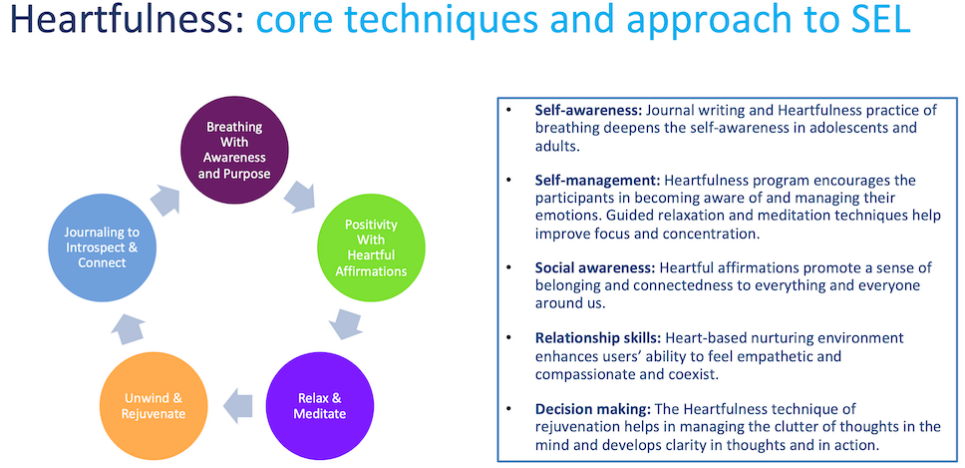
Heartfulness approach to social-emotional learning [15]. SEL: social-emotional learning.

HeartBot is an iOS app developed by high school students for users 14 years and older to learn and practice the 5 practical Heartfulness tools: relaxation, meditation, rejuvenation, affirmations, and breathing [14]. This app expands upon previous social-emotional wellness apps by implementing these tools and journal writing to promote the development of the 5 core competencies of social and emotional learning, as charted by the Collaborative for Academic, Social, and Emotional Learning [15] (Figure 3).

### Procedures

A total of 88 participants of mixed ages who were 14 years and older took part in the study. Participants, who were screened to fit the inclusion criteria, were asked to provide a written consent (18 years or older) or assent form (younger than 18 years). They were randomly divided into the HeartBot and the waitlist control groups using an online random name generator to balance the two groups by age. There was no significant difference in the number of people in the two groups. Participants randomized to the waitlist group received the HeartBot intervention after the waitlist period.

The HeartBot group downloaded the app and used it daily for 21 days based on a calendar that provided them with step-by-step outlines of the tools for each day (Multimedia Appendix 1). The control group did not download the app and experienced no change to their daily routines. Presurveys were sent to all participants via email before and after their 21-day challenge. Data collected gave a baseline and final quantitative measure of participants’ stress and emotional wellness. Participants were not individually monitored or targeted during their participation. Those who completed the challenge were entered in a raffle and had a chance to win one of 10 US $50 gift cards.

### Sample Size

A sample size larger than 30 and less than 500 is appropriate for most research. With a 95% confidence level and a 9.5% margin of error, the sample size came to 100. We also noted that estimating a margin of error for sample sizes ranging from 10 to 10,000 reduces to 10% [16].

### Measures

Participant stress levels were measured using the Perceived Stress Scale (PSS). This 10-item scale measures the degree to which a situation in someone’s life is considered stressful [17]. The survey consists of a 5-point scale for each question, where 0=never and 4=very often, to indicate how often respondents felt a certain way about a certain stimulus or event. The minimum score is 0 and the maximum score is 40, with higher scores suggesting higher stress levels. This scale has been proven valid through its use in other studies, which found that higher PSS scores are associated with a greater vulnerability to stressful life event–elicited depressive symptoms [17]. Similarly, reliability between the collected PSS scores and PSS data norms was about 0.78, indicating a very reliable measure.

The emotional wellness of the participants was measured using the EPOCH (Engagement, Perseverance, Optimism, Connectedness, and Happiness) Measure of Adolescent Well-Being. This is a 20-item scale used to measure 5 different positive characteristics: engagement, perseverance, optimism, connectedness, and happiness [18]. Each question on this survey consists of a 5-point scale, where “almost never” or “not at all like me” is a 1 and “almost always” or “very much like me” is a 5. On this scale, the minimum score is 20 and the maximum score is 100, and higher scores correlate with a higher level of emotional wellness. The test has demonstrated very high validity and reliability, with a Cronbach α of .90, Guttman λ6 of 0.91, minimum split-half reliability of 0.75, and maximum split-half reliability of 0.93 [18].

### Data Analysis

The data collected online from the pre- and postsurveys were cleaned and adjusted to eliminate data errors or corruption. Then, the dependent variables were examined for normality and missing values. In examining the relationship between variables, researchers can use a *t* test or analysis of variance (ANOVA) to compare the means of two groups on the dependent variable [19]. Baseline differences from the presurvey scores between the HeartBot and the control groups on the PSS and EPOCH were measured. Descriptive statistics and the difference between the pre- and postsurvey scores from each of the outcome measures (PSS, EPOCH) were analyzed. Statistical significance was determined by *P*<.05.

## Results

### Overview

A total of 135 participants were recruited for the study. Of these, 88 participants (65.2%) completed the full 21-day challenge, and the data were analyzed. A total of 46 participants in the HeartBot group and 42 participants in the control waitlisted group completed the pre- and postsurveys. Out of the 46 participants in the intervention group, 20 (43%) were aged between 14 and 17 years and 26 (57%) were older than 18 years.

### Baseline Equivalences

Baseline level as prescores and postscores after the 21-day challenge reflected the outcome measures for changes in the mean scores in the perceived stress levels and the 5 characteristics for well-being in the EPOCH scores. The initial analysis examined the differences in the PSS and EPOCH baseline scores in the HeartBot and control group. The baseline equivalence compared the average baseline characteristics for the HeartBot and control groups.

There was no significant difference between the baseline mean PSS scores in the HeartBot group and the control group at the beginning of the study, as shown by 2-tailed paired *t* tests. Although the participants’ selection in the two groups was randomized, there were significant differences at baseline in the EPOCH scores between the HeartBot and the control groups (Multimedia Appendix 2).

### Descriptive Analysis

**Figure 4:**
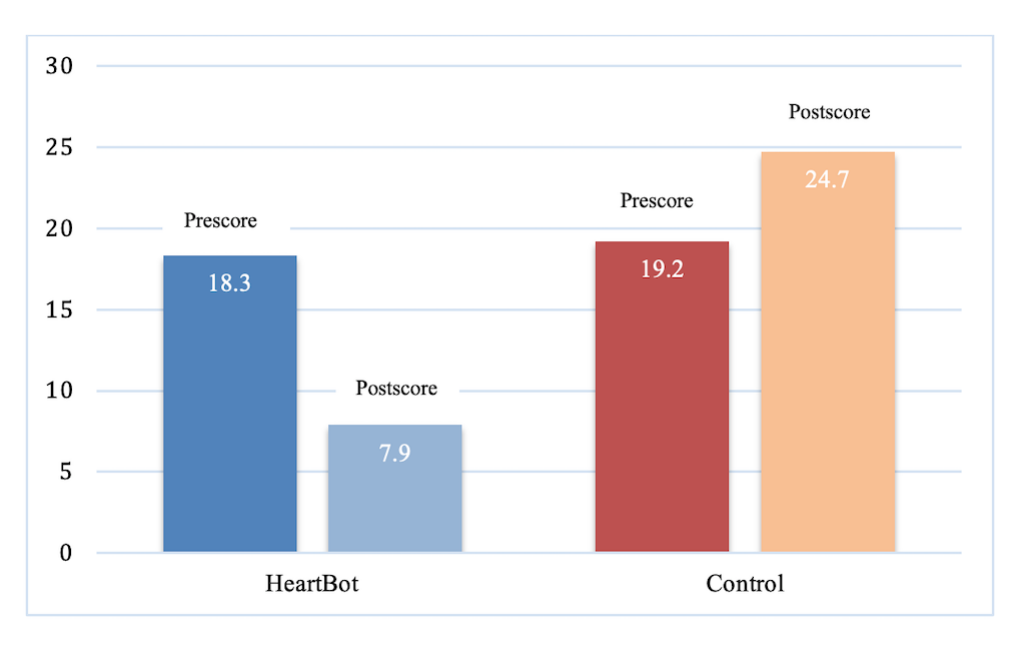
Change in Perceived Stress Scale mean scores on pre- and postsurveys for the HeartBot and control groups.

The mean PSS pre- and postscores in the HeartBot group showed a significant decrease from 18.3 to 7.89 (*P*<.001). In contrast, the mean scores increased from 19.2 to 24.7 (*P*<.001) in the control group (Figure 4). This finding suggests a significant decrease in the perceived stress in the HeartBot group compared with the participants in the control group (Multimedia Appendix 3).

One-way ANOVA examined the difference between the mean PSS score in the HeartBot and control groups. The ANOVA was significant (*F*_1,82_=125.76, *P*<.001). This result supports the conclusion that there is a statistically significant and robust relationship between the two groups and the perceived stress scales (Multimedia Appendix 3).

**Figure 5:**
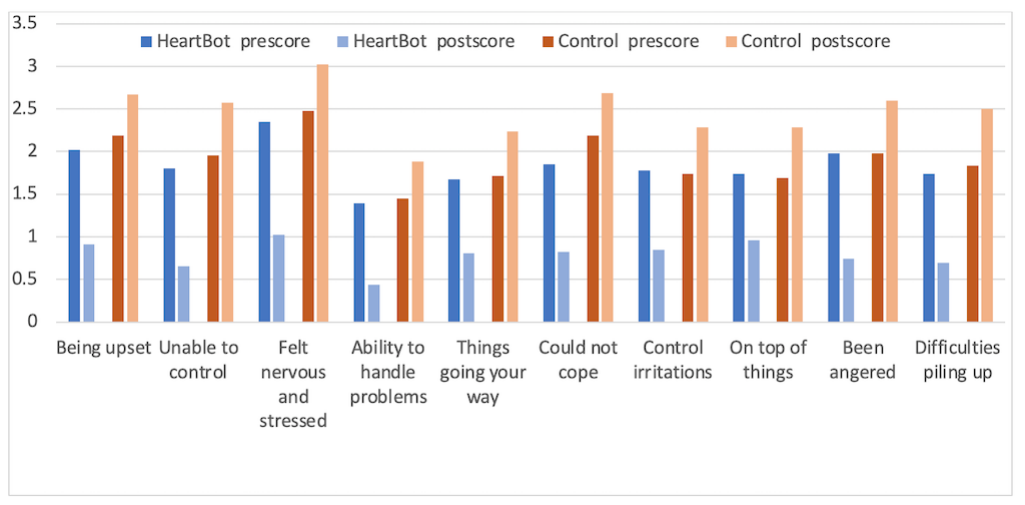
Summary of statistics on items on the Perceived Stress Scale survey.

The questions (items 1-10) on the PSS, which ask participants if they were feeling nervous and stressed (question 3), could not cope (question 6), and have been angered (question 9), showed a decreased score in HeartBot participants, indicating that the app helped in reducing stress. Figure 5 shows the responses to the questions on participants’ ability to handle problems (question 4) and things going their way (question 5), indicating an improvement in coping skills in the HeartBot group. As noted, for the HeartBot group, PSS scores increased for items that showed stress management and coping, showing an improvement. This finding indicates that the app worked on the participants in that group and that HeartBot helped the participants reduce perceived stress.

**Figure 6:**
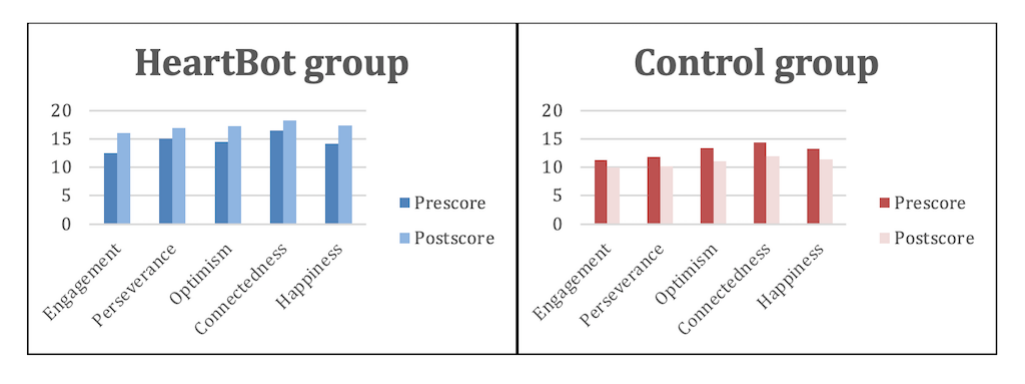
Change in EPOCH total mean scores on the pre- and postsurveys for the two groups. EPOCH: Engagement, Perseverance, Optimism, Connectedness, and Happiness.

The descriptive data and comparison of the means in the HeartBot group for the 5 characteristics of EPOCH showed an increase in all 5 positive characteristics (Figure 6). The findings indicate that the positive characteristics of perseverance, optimism, and connectedness increased significantly in the HeartBot group. There was also a decrease noted in all 5 positive characteristics in the control group.

For the postintervention total EPOCH scores, the median total EPOCH score was 89.0 for the HeartBot group and 60.5 for the control group. There was a significant difference in the medians of the postintervention total EPOCH scores for the HeartBot and control groups, with Kruskal-Wallis χ^2^_1_=51.0 (*P*<.001) and Bonferroni correction 9.5 × 10^–13^.

In comparing the baseline and postintervention total EPOCH scores for the HeartBot group, there was a significant difference in the medians of the total scores (*W*=411.5, *P*<.001). In comparing the baseline and postintervention total EPOCH scores for the control group, there was a significant difference in the medians of the total scores (*W*=1145, *P*=.01). Paired 2-tailed *t* tests on baseline pre-EPOCH and post-EPOCH scores between the HeartBot and control groups showed a significant difference (Multimedia Appendix 4).

### Age Categories

An adult is any participant aged 18 years or older, and a minor is any participant younger than 18 years (14-17 years for this study).

For adults, a statistically significant difference was found on the PSS at baseline in contrast to the postdata (HeartBot group: t_26_=7.14, *P*<.001; control group: t_27_=–4.07, *P*<.001). When comparing the data for minors, there was a statistically significant difference found on the PSS at baseline in contrast to the postdata (HeartBot group: t_20_=8.61, *P*<.001; control group: t_15_=–2.8, *P*<.001). This finding suggests that there was a significant decrease in perceived stress in the HeartBot group in comparison with the control group.

For adults, comparing the baseline and postintervention total EPOCH scores for the HeartBot group showed that there was a significant difference in the medians of the total scores (*W*=130.5, *P*<.001). When comparing the baseline and postintervention total EPOCH scores for adults in the control group, there was no significant difference in the medians of the total scores (*W*=434.5, *P*=.23). For minors, comparing the baseline and postintervention total EPOCH scores revealed a significant difference in the medians of the total scores for the HeartBot group (*W*=75, *P*<.001) and for the control group (*W*=186, *P*=.002).

## Discussion

### Principal Findings

This study’s objectives were to assess changes in scores measuring stress levels (using PSS) and emotional wellness (using EPOCH). This study showed that there was a significant decrease in the perceived stress levels and an increase in the emotional well-being of participants who used the app for 21 days for 30 minutes every day based on a 21-day calendar. Apps have been shown to provide more efficient delivery of health care and increase treatment effectiveness [20]. This app provides users with a convenient way to practice something that would be harder for them to do otherwise. This study demonstrated that the HeartBot app reduces stress levels and improves emotional well-being for its users.

A previous study on Heartfulness in schools showed a significant decrease in stress levels and a significant increase in participants’ overall well-being [12]. Another study showed that practicing Heartfulness techniques for 12 weeks demonstrated improved wellness and amelioration of burnout [21]. The results of this study are consistent with these studies.

The findings from this study contribute to the growing movement of stress management apps and their effectiveness. Small to moderate effects on global well-being and positive affect were seen over 1 working day of using a mindfulness app [22]. Combining technology and mindfulness techniques has been shown to elicit meaningful benefits by increasing the accessibility and efficacy of mindfulness training [23]. Teenagers prefer using a digital medium for help rather than a face-to-face approach [24]. Studies have shown that computerized platforms such as apps are a comparable and valid means of delivering mindfulness training compared with face-to-face interactions, showing that apps can be just as effective a method of practicing mindfulness [25]. This study adds to the evidence that the HeartBot app can help significantly decrease stress and improve emotional wellness.

This study addresses how much time a user needs to spend on the app to reap its benefits. All participants in the HeartBot group spent 30 minutes a day for 21 days and saw a significant difference in their stress levels. EPOCH showed a significant increase in the HeartBot group for perseverance (*P*=.009), optimism (*P*=.005), and connectedness (*P*<.001). Future research could further explore the social benefits of Heartfulness and the long-term effects of using the HeartBot app. Notably, studies in the future could explore the connection between loneliness and the practice of Heartfulness tools.

Going into this study, we expected to see a significant result for the group of participants aged 14 to 18, as teenagers are more likely to prefer using technological mediums than are older participants [24]. The app was designed specifically with this age cohort in mind. To conclude, the results suggest a significant decrease in the perceived stress levels and a significant increase in social-emotional well-being for all users aged 14 years or older. Future studies can be designed to investigate the effect of HeartBot after 3 months and then after 6 months to provide further evidence.

### Limitations

The current study is the first study to explore the effectiveness of the HeartBot app. Some limitations of this study were identified. First, participants used the app for 21 days based on a calendar, but the specific data reporting the exact length of time that each participant spent on the app were not measured. Second, this study had restrictions on time and resources. Future studies could use a randomized controlled design with a larger sample and longer duration to establish the app’s effectiveness. Third, although groups were randomized and no self-selection was involved, there were some baseline differences in the EPOCH scores between the two groups. Fourth, 27% (24/88) of the sample participants were men and 73% (64/88) were women, limiting generalizability to men. Fifth, as the study included minors and adults, further studies could focus on one target group for more substantial data. Sixth, EPOCH has demonstrated good reliability, validity, and sensitivity to change in adolescents but not in adults. Future work should extend the findings of this feasibility study and consider these limitations to lead to broader generalizability.

### Implications for Mental Health

The authors examined the HeartBot app’s effectiveness in reducing perceived stress and improving overall well-being in participants 14 years and older. These findings provide evidence that HeartBot enables users who use the app for 21 days for 30 minutes every day to manage stress effectively by providing personalized guided audios and Heartfulness tools. Results from the quantitative analyses provide further evidence supporting the use of this app for providing a convenient way for adolescents and adults to learn and practice Heartfulness tools for stress management and social-emotional well-being. More widespread usage of the app under this study could be encouraged based on this preliminary evidence of its effectiveness.

## Appendix

Multimedia Appendix 1 Calendar for HeartBot study.

Multimedia Appendix 2 Statistical data for baseline equivalence.

Multimedia Appendix 3 Statistical data for ANOVA- Single Factor- HeartBot and Control- Post-PSS scores.

Multimedia Appendix 4 Statistical data for HeartBot and Control- Post-EPOCH scores.

## Abbreviations

ANOVA: analysis of variance
EPOCH: Engagement, Perseverance, Optimism, Connectedness, and Happiness
PSS: Perceived Stress Scale
