# Supplementary material for: A Relaxation App (HeartBot) for Stress and Emotional Well-Being Over a 21-Day Challenge: Randomized Survey Study
Source: JMIR Form Res. 2021 Jan 29;5(1):e22041. doi: 10.2196/22041 (PMC7880805; doi:10.2196/22041)
Supplement: Multimedia Appendix 1 [file formative_v5i1e22041_app1.pdf]

21 Day Calendar

**Day 1**<br><br>1. 6 min Relaxation<br>2. 10 min Meditation<br>3. 7 min PEMS Journaling<br>4. 7 min Gratitude Journaling	**Day 2**<br><br>1. 6 min Breathe<br>2. 10 min Meditation<br>3. 7 min PEMS Journaling<br>4. 7 min Gratitude Journaling	**Day 3**<br><br>1. 6 min Affirm<br>2. 10 min Meditation<br>3. 7 min PEMS Journaling<br>4. 7 min Gratitude Journaling	**Day 4**<br><br>1. 6 min Unwind<br>2. 10 min Meditation<br>3. 7 min PEMS Journaling<br>4. 7 min Gratitude Journaling
**Day 5**<br><br>1. 6 min Relaxation<br>2. 10 min Meditation<br>3. 7 min PEMS Journaling<br>4. 7 min Gratitude Journaling	**Day 6**<br><br>1. 6 min Breathe<br>2. 10 min Meditation<br>3. 7 min PEMS Journaling<br>4. 7 min Gratitude Journaling	**Day 7**<br><br>1. 6 min Affirm<br>2. 10 min Meditation<br>3. 7 min PEMS Journaling<br>4. 7 min Gratitude Journaling	**Day 8**<br><br>1. 6 min Unwind<br>2. 10 min Meditation<br>3. 7 min PEMS Journaling<br>4. 7 min Gratitude Journaling
**Day 9**<br><br>1. 6 min Relaxation<br>2. 10 min Meditation<br>3. 7 min PEMS Journaling<br>4. 7 min Gratitude Journaling	**Day 10**<br><br>1. 6 min Breathe<br>2. 10 min Meditation<br>3. 7 min PEMS Journaling<br>4. 7 min Gratitude Journaling	**Day 11**<br><br>1. 6 min Affirm<br>2. 10 min Meditation<br>3. 7 min PEMS Journaling<br>4. 7 min Gratitude Journaling	**Day 12**<br><br>1. 6 min Unwind<br>2. 10 min Meditation<br>3. 7 min PEMS Journaling<br>4. 7 min Gratitude Journaling

**Day 13**<br><br>1. 6 min Relaxation<br>2. 10 min Meditation<br>3. 7 min PEMS Journaling<br>4. 7 min Gratitude Journaling	**Day 14**<br><br>1. 6 min Breathe<br>2. 10 min Meditation<br>3. 7 min PEMS Journaling<br>4. 7 min Gratitude Journaling	**Day 15**<br><br>1. 6 min Affirm<br>2. 10 min Meditation<br>3. 7 min PEMS Journaling<br>4. 7 min Gratitude Journaling	**Day 16**<br><br>1. 6 min Unwind<br>2. 10 min Meditation<br>3. 7 min PEMS Journaling<br>4. 7 min Gratitude Journaling
**Day 17**<br><br>1. 6 min Relaxation<br>2. 10 min Meditation<br>3. 7 min PEMS Journaling<br>4. 7 min Gratitude Journaling	**Day 18**<br><br>1. 6 min Breathe<br>2. 10 min Meditation<br>3. 7 min PEMS Journaling<br>4. 7 min Gratitude Journaling	**Day 19**<br><br>1. 6 min Affirm<br>2. 10 min Meditation<br>3. 7 min PEMS Journaling<br>4. 7 min Gratitude Journaling	**Day 20**<br><br>1. 6 min Unwind<br>2. 10 min Meditation<br>3. 7 min PEMS Journaling<br>4. 7 min Gratitude Journaling
**Day 21**<br><br>1. 6 min Relaxation<br>2. 10 min Meditation<br>3. 7 min PEMS Journaling<br>4. 7 min Gratitude Journaling			
