# Supplementary material for: A Relaxation App (HeartBot) for Stress and Emotional Well-Being Over a 21-Day Challenge: Randomized Survey Study
Source: JMIR Form Res. 2021 Jan 29;5(1):e22041. doi: 10.2196/22041 (PMC7880805; doi:10.2196/22041)
Supplement: Multimedia Appendix 2 [file formative_v5i1e22041_app2.docx]

# Supplementary data 1. Paired t-Test on Baseline PSS Scores between HeartBot and Control groups

|  | HeartBot (HB) (n=46) | Control (C) (n=42) |
| --- | --- | --- |
|  | *PrePSS-HB* | *PrePSS-C* |
| Mean | 18.30952381 | 19.21428571 |
| Variance | 44.26771196 | 30.95296167 |
| Observations | 42 | 42 |
| Pearson Correlation | 0.164866949 |  |
| Hypothesized Mean Difference | 0 |  |
| df | 41 |  |
| t Stat | -0.738646494 |  |
| *P*(T<=t) one-tail | 0.232164641* |  |
| t Critical one-tail | 1.682878002 |  |
| *P*(T<=t) two-tail | 0.464329283* |  |
| t Critical two-tail | 2.01954097 |  |

*PSS* Perceived Stress Scale

There was no significant difference between the baseline mean scores for the PSS of the HeartBot group and the control group at the beginning of the study, as shown by paired t-tests. The mean score of PSS for the baseline HeartBot group was about 18.3095, and the baseline for the control group was about 19.2143. The *P* value for this data, *P*=.23 (*NS*), shows that although baseline (pre) is higher for HeartBot than for the control group, the difference is not significant.

# Supplementary data 2. Paired t-Test on Baseline EPOCH Scores between HeartBot and Control groups

|  | HeartBot (HB) (n=46) | Control (C) (n=42) |
| --- | --- | --- |
|  | *PreEPOCH- HB* | *PreEPOCH- C* |
| Mean | 73.0952381 | 66.14285714 |
| Variance | 190.8687573 | 239.1986063 |
| Observations | 42 | 42 |
| Pearson Correlation | -0.033054082 |  |
| Hypothesized Mean Difference | 0 |  |
| df | 41 |  |
| t Stat | 2.137826466 |  |
| *P*(T<=t) one-tail | 0.019270334* |  |
| t Critical one-tail | 1.682878002 |  |
| *P*(T<=t) two-tail | 0.038540669* |  |
| t Critical two-tail | 2.01954097 |  |

*EPOCH* The EPOCH Measure of Adolescent Well-being

Total scores, the sum of all responses to all questions for each participant for EPOCH, were used to determine the baseline equivalence. For the baseline total EPOCH scores for the two groups, the median total EPOCH score is 73.0 for the HeartBot group and 67.5 for the control group. However, Kruskal-Wallis non-parametric tests indicated a significant difference in the medians of the baseline total EPOCH scores for the HeartBot and control groups with Kruskal-Wallis chi-squared = 3.6181, df = 1, *P*=.057. The *P* values were adjusted using BH and Bonferonni, and there was no significance. For Kruskal-Wallis, the effect size was small, indicated by an epsilon squared value of .0416, which means that the likelihood of scores is greater in one group than the other is small. Although the participants’ selection in the two groups was randomized, there were significant differences at baseline for EPOCH scores between the HeartBot and the control groups
