# Supplementary material for: A Relaxation App (HeartBot) for Stress and Emotional Well-Being Over a 21-Day Challenge: Randomized Survey Study
Source: JMIR Form Res. 2021 Jan 29;5(1):e22041. doi: 10.2196/22041 (PMC7880805; doi:10.2196/22041)
Supplement: Multimedia Appendix 3 [file formative_v5i1e22041_app3.docx]

# Supplementary data 3. Paired t-Tests on baseline pre-PSS and post-PSS Scores between HeartBot and Control groups

|  | HeartBot (HB) (n=46) | | Control (C) (n=42) | |
| --- | --- | --- | --- | --- |
|  | *PrePSS-HB* | *PostPSS-HB* | *PrePSS-C* | *PostPSS-C* |
| Mean | 18.32608696 | 7.891304348 | 19.21428571 | 24.73809524 |
| Variance | 44.71352657 | 37.16570048 | 30.95296167 | 70.53948897 |
| Observations | 46 | 46 | 42 | 42 |
| Pearson Correlation | 0.596166338 |  | 0.537297681 |  |
| Hypothesized Mean Difference | 0 |  | 0 |  |
| df | 45 |  | 41 |  |
| t Stat | 12.26912343 |  | -4.999075834 |  |
| *P*(T<=t) one-tail | 2.95716E-16* |  | 5.62478E-06* |  |
| t Critical one-tail | 1.679427393 |  | 1.682878002 |  |
| *P*(T<=t) two-tail | 5.91432E-16* |  | 1.12496E-05* |  |
| t Critical two-tail | 2.014103389 |  | 2.01954097 |  |

A statistically significant difference was found on the PSS at baseline **compared** to post-data, t (46) = 12.27, *P*<.001 for the HeartBot group. In contrast, there was a statistically significant difference found on the PSS at baseline in **comparison** to post-data, t (42) = -4.99, *P*<.001 for the control group.

# Supplementary data 4. ANOVA: Single Factor- HeartBot and Control: Post-PSS scores

| SUMMARY |  |  |  |  |  |  |
| --- | --- | --- | --- | --- | --- | --- |
| *Groups* | *Count* | *Sum* | *Average* | *Variance* |  |  |
| HeartBot | 42 | 439 | 10.452381 | 33.9610918 |  |  |
| Control | 42 | -232 | -5.5238095 | 51.2799071 |  |  |
| ANOVA |  |  |  |  |  |  |
| *Source of Variation* | *SS* | *df* | *MS* | *F* | *P-value* | *F crit* |
| Between Groups | 5360.011905 | 1 | 5360.0119 | 125.761358 | 3.1305E-18* | 3.95738832 |
| Within Groups | 3494.880952 | 82 | 42.6204994 |  |  |  |
| Total | 8854.892857 | 83 |  |  |  |  |
