# Supplementary material for: A Relaxation App (HeartBot) for Stress and Emotional Well-Being Over a 21-Day Challenge: Randomized Survey Study
Source: JMIR Form Res. 2021 Jan 29;5(1):e22041. doi: 10.2196/22041 (PMC7880805; doi:10.2196/22041)
Supplement: Multimedia Appendix 4 [file formative_v5i1e22041_app4.docx]

# Supplementary data 5. Paired t-Tests on baseline pre-EPOCH and post-EPOCH Scores between HeartBot and Control groups

|  | HeartBot (HB) (n=46) | | Control (C) (n=42) | |
| --- | --- | --- | --- | --- |
|  | *PreEPOCH- HB* | *PostEPOCH-HB* | *PreEPOCH- C* | *PostEPOCH- C* |
| Mean | 72.73913043 | 87.41304348 | 66.14285714 | 55.97619048 |
| Variance | 178.3304348 | 102.4700483 | 239.1986063 | 362.3164925 |
| Observations | 46 | 46 | 42 | 42 |
| Pearson Correlation | 0.561385848 |  | 0.686424491 |  |
| Hypothesized Mean Difference | 0 |  | 0 |  |
| df | 45 |  | 41 |  |
| t Stat | -8.761703107 |  | 4.689987591 |  |
| *P*(T<=t) one-tail | 1.37922E-11* |  | 1.51119E-05* |  |
| t Critical one-tail | 1.679427393 |  | 1.682878002 |  |
| *P*(T<=t) two-tail | 2.75844E-11* |  | 3.02237E-05* |  |
| t Critical two-tail | 2.014103389 |  | 2.01954097 |  |

For the post-intervention total EPOCH scores, the median total EPOCH score is 89.0 for HeartBot and 60.5 for the Control group. There was a significant difference in the medians of the post-intervention total EPOCH scores for the HeartBot and control groups with Kruskal-Wallis chi-squared = 50.998, df = 1, *P*<.001 and with Bonferonni correction 9.5e-13. The effect size was large, as indicated by an epsilon squared value of .586. With the large effect size, there is a greater likelihood that scores are higher than others.

In comparing the baseline and post-intervention total EPOCH scores for the HeartBot group, there was a very significant difference in the medians of the total scores (W = 411.5, *P*<.001). The Cohen d effect size (Z/sqrt (sample size)) is 0.7441995 indicating a large effect size. In comparing the baseline and post-intervention total EPOCH scores for the control group, there was a significant difference in the medians of the total scores (W = 1145, *P*=.012). The Cohen d effect size (Z/sqrt (sample size)) is 0.3625644 indicating a moderate effect size. Paired t-Tests on baseline pre-EPOCH and post-EPOCH Scores between HeartBot and Control groups show a significant difference.

Age Categories

For adults, a statistically significant difference was found on the PSS at baseline in contrast to post-data, t (26) = 7.14, *P*<.001 for the HeartBot group, and the control group t (27) = -4.07, *P*<.001. In comparing the data for minors, there was a statistically significant difference found on the PSS at baseline in contrast to post-data, t (20) = 8.61, *P*<.001 for the HeartBot group and the control group t (15) = -2.8, *P*<.001. This finding suggests that there was a significant decrease in the perceived stress in the HeartBot group in comparison to the participants in the control group.

For adults, in comparing the baseline and post-intervention total EPOCH scores for the HeartBot group, there was a very significant difference in the medians of the total scores (W = 130.5, *P*<.001). The Cohen d effect size (Z/sqrt (26)) is .7434479 indicating a large effect size. In comparing the baseline and post-intervention total EPOCH scores for adults’ control group, there was no significant difference in the medians of the total scores (W = 434.5, *P*=.2289). However, Cohen d effect size (Z/sqrt (27)) is .3106879 indicating a moderate effect size. For minors, in comparing the baseline and post-intervention total EPOCH scores for the HeartBot group, there was a significant difference in the medians of the total scores (W = 75, *P*<.001). The Cohen d effect size (Z/sqrt (20)) is .7535083 indicating a large effect size. In comparing the baseline and post-intervention total EPOCH scores for the Control group, there was a significant difference in the medians of the total scores (W = 186, *P*=.002). The Cohen d effect size (Z/sqrt (15)) is .7827585 indicating a large effect size.
